# Molecular basis for convergent evolution of glutamate recognition by pentameric ligand-gated ion channels

**DOI:** 10.1038/srep08558

**Published:** 2015-02-24

**Authors:** Timothy Lynagh, Robin N. Beech, Maryline J. Lalande, Kevin Keller, Brett A. Cromer, Adrian J. Wolstenholme, Bodo Laube

**Affiliations:** 1 Neurophysiology and Neurosensory Systems, Technical University of Darmstadt, Darmstadt 64287, Germany; 2 Institute of Parasitology, McGill University, Macdonald Campus, St Anne-de-Bellevue, QC, Canada; 3 Health Innovations Research Institute, School of Medical Sciences, RMIT University, Bundoora, VIC, Australia; 4 Department of Infectious Diseases and Center for Tropical and Emerging Global Diseases, University of Georgia, Athens, GA, USA

## Abstract

Glutamate is an indispensable neurotransmitter, triggering postsynaptic signals upon recognition by postsynaptic receptors. We questioned the phylogenetic position and the molecular details of when and where glutamate recognition arose in the glutamate-gated chloride channels. Experiments revealed that glutamate recognition requires an arginine residue in the base of the binding site, which originated at least three distinct times according to phylogenetic analysis. Most remarkably, the arginine emerged on the principal face of the binding site in the Lophotrochozoan lineage, but 65 amino acids upstream, on the complementary face, in the Ecdysozoan lineage. This combined experimental and computational approach throws new light on the evolution of synaptic signalling.

Rapid conversion of chemical to electrical signals at the cell membrane is a hallmark of the animal nervous system. This process is mediated by ligand-gated ion-channels (LGICs), membrane-spanning channels that open rapidly upon recognition of a specific transmitter (agonist). Gating of excitatory, cation-selective LGICs leads to membrane depolarization, increased neuronal signalling and muscle contraction. Conversely, gating of inhibitory, anion-selective LGICs generally leads to membrane hyperpolarization, inhibiting responses to synaptic activity. Both cation- and anion-selective pentameric LGICs (pLGICs) are descended from an ancestral pLGIC present before the prokaryote-eukaryote dichotomy^1^. Cation-selective pLGICs typically respond to acetylcholine, whereas the anion-selective pLGICs respond to an especially wide variety of agonists, predominantly GABA and glycine in vertebrates and in addition, glutamate and biogenic amines in invertebrates^2^,^3^. Characteristics of the original anion-selective pLGIC and the molecular details of how the current repertoire of inhibitory signalling arose remains an open question that has major implications for our understanding of the evolution of animal neuromuscular control and development^4^.

In the case of glutamate, functional homo-pentameric glutamate-gated chloride channels (GluCls) have been characterized in the Ecdysozoan^5^,^6^,^7^,^8^ and Molluscan lineages^9^. Phylogenetic analysis suggested that these may either be descended from a single ancient glutamate receptor that subsequently diversified to also produce receptors responding to other neurotransmitters or, more likely, represent descendants from distinct origins that independently converged on glutamate binding^9^. More recently, the characterization of several homomeric GluCls in the Platyhelminth, *Schistosoma mansoni*^10^, supported the idea that glutamate-binding pLGICs are ancient but could not provide additional clarification of a single or multiple origins. The inference that glutamate recognition has multiple origins is based largely on experimental verification of GluCls in a few species, coupled with the assumption that GluCl subunits form well-defined phylogenetic clades that are likely to respond to the same agonist. As such, the hypothesis proposed by Kehoe *et al*.^9^ of multiple origins for glutamate recognition awaits a mechanistic insight. We have taken the approach that identifying specific functional amino acids essential for glutamate binding to GluCls will improve the precision with which we can identify the phylogenetic position of when and where glutamate recognition arose.

The binding of glutamate to the GluCl closely reflects that of the other amino acid agonists, GABA and glycine, to their receptors (GABA_A_Rs and GlyRs; reviewed recently^11^): the agonist amine is coordinated by the “aromatic box” of the principal subunit, at one side of the intersubunit interface; and the agonist carboxyl (one of two for glutamate) binds to positively charged and hydroxyl side chains of the complementary subunit at the opposing side of the interface. The aromatic box and the positively charged and hydroxyl side chains (hereafter referred to as the amino acid agonist motif, “AAA motif”) are highly conserved in agonist-binding GluCl, GlyR and GABA_A_R subunits, in which the mutation of these residues greatly reduces agonist potency^12^,^13^,^14^,^15^,^16^,^17^. Glutamate, however, is unique in that its recognition also requires the coordination of an additional carboxyl moiety. Here, we identify the amino acids of Ecdysozoan, Molluscan and Platyhelminth GluCls that underlie the coordination of this carboxyl moiety, using a combined computational, experimental and phylogenetic approach. These results confirm that glutamate recognition by the pLGIC family has evolved at least three times, with discrete parts of receptor sequence utilized for carboxyl-binding in the Lophotrochozoan and Ecdysozoan lineages.

## Results

### Putative determinants of glutamate recognition in diverse GluCls

**Figure 1:**
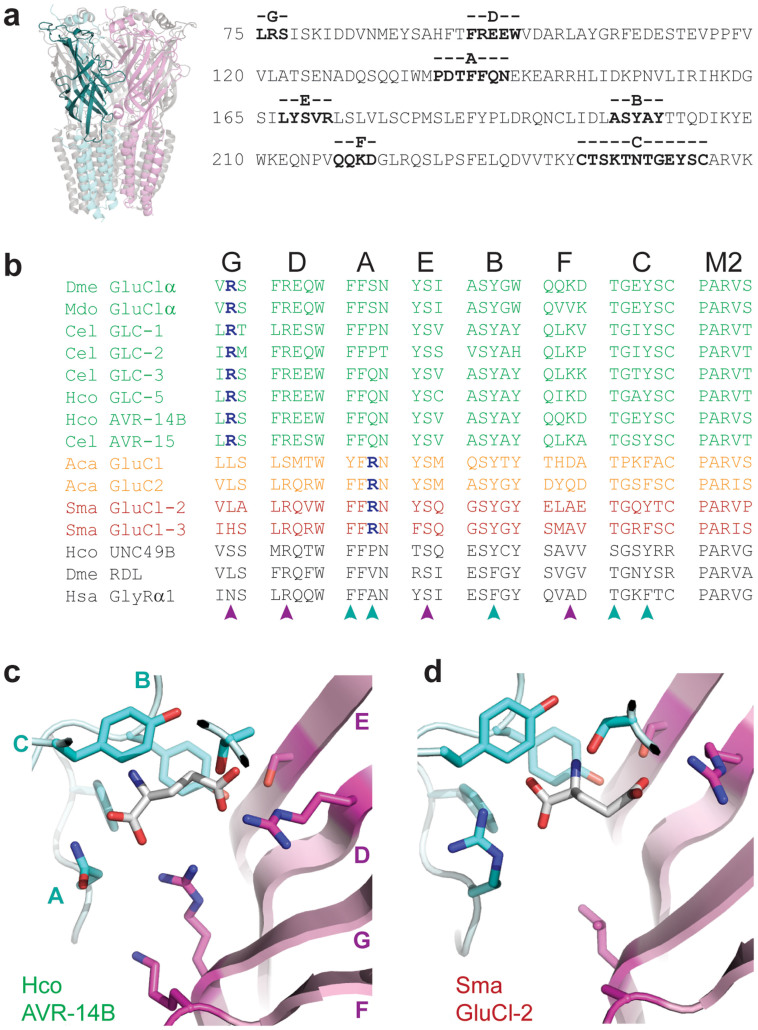
Molecular determinants of glutamate recognition by diverse GluCls. (a) GluCl architecture. Homology model of the homo-pentameric AVR-14B GluCl, viewed from within the membrane plane. Two subunits are in cyan and magenta, with a single subunit's extracellular domain in bold. The amino acid sequence of this extracellular domain is shown to indicate the positions of agonist-binding loops A-G. (b) Partial amino acid sequence alignment of verified ecdysozoan (green), molluscan (orange) and platyhelminth (red) GluCl subunits. Two ecdysozoan GABA_A_R subunits and one vertebrate GlyR subunit are also shown (black). Only the ligand-binding loops A-G and part of the chloride channel-forming M2 helix are shown. Dme, *Drosophila melanogaster*; Mdo, *Musca domestica*; Cel, *Caenorhabditis elegans*; Hco, *Haemonchus contortus*; Aca, *Aplysia californica*; Sma, *Schistosoma mansoni*; Hsa, *Homo sapiens*. Arrows indicate agonist-binding residues of the AAA motif or the critical arginine residues (blue, bold), all of which are illustrated in panels (c) and (d) and discussed at length in main text. For original references, containing sequence and functional information on each subunit, see *Methods; Sequence alignments and computational dockings*. (c) Highest-ranked computational docking of glutamate to the ecdysozoan AVR-14B GluCl model, showing Loops A-C of the principal face from one subunit (cyan) and D-G of the complementary face from an adjacent subunit (magenta). One Loop G arginine η nitrogen atom is 2.4 Å from one agonist α-carboxyl oxygen atom. In panels (c) and (d), most of Loop C is removed for clarity. (d) Highest-ranked docking of glutamate to the platyhelminth SmGluCl-2 model. Loop A arginine ε and η nitrogen atoms are 2.8 and 3.2 Å from agonist α-carboxyl oxygen atoms, respectively.

Alignment of Arthropod, Nematode, Mollusk and Platyhelminth sequence from subunits that form experimentally verified GluCls revealed a highly conserved motif responsible for glutamate recognition. This includes the AAA motif, comprising three aromatic and one threonine residue from principal Loops A, B and C (cyan arrows, Fig. 1b) and one arginine and one serine residue from complementary Loops D and E (magenta arrows, Fig. 1b). This motif is present in essentially all GluCls, suggesting that the mode in which glutamate binds is similar in all GluCls, with the amino nitrogen coordinated at the principal face in the aromatic box and the γ-carboxyl group coordinated at the complementary face by the Loop D arginine and Loop C and E hydroxyl side chains. This interpretation is supported by the fact that in GluCls, GABA_A_Rs and GlyRs that share the AAA motif, agonists bind in the same orientation, according to structural studies^18^, homology modelling^16^,^19^ and mutagenesis data^12^,^13^,^14^,^15^,^16^,^19^,^20^,^21^. According to the structure of the glutamate-bound GLC-1 GluCl^18^ from *C. elegans*, the coordination of the additional α-carboxyl moiety of glutamate is achieved by positively charged Loop F lysine and Loop G arginine side chains, present in this GluCl but absent from GABA_A_Rs and GlyRs^18^. Our alignment showed that the Loop F lysine is moderately conserved but the Loop G arginine is absolutely conserved in ecdysozoan GluCls (Fig. 1b). The critical role of the Loop G arginine is supported by mutagenesis in GLC-2 from *C. elegans*^14^. These positively charged side chains are not conserved, however, in lophotrochozoan GluCl subunits (Fig. 1b). This raises the question as to how lophotrochozoan GluCls accommodate the glutamate α-carboxyl, or if indeed glutamate binds in the same mode to these channels.

To address these questions, we used Modeller software^22^ to build homology models of the AVR-14B GluCl from the nematode *Haemonchus contortus* and the SmGluCl-2 from the platyhelminth *S. mansoni*, based on the *C. elegans* GluCl structural template^18^, as described previously^23^. The reliability of the models is enhanced by high sequence identity (>40%) with the template, particularly in the agonist-binding loops (Fig. 1b). *In silico* docking of glutamate to the agonist-binding site of these models, using Autodock Vina^24^, indicated that glutamate interacted with the AAA motif similarly in both models (Fig. 1c, d), supporting the notion that the binding mode is common to all GluCls. There is, however, a striking difference between Ecdysozoan and Lophotrochozoan GluCl interactions with the glutamate α-carboxyl. In the absence of a positively charged side chain in Loops F or G of the complementary face of SmGluCl-2, an arginine side chain from Loop A of the principal face approaches the glutamate α-carboxyl to within 2.8 Å (Fig. 1d). This position is occupied by an arginine in all Lophotrochozoan GluCl sequences, whereas it is occupied by serine, proline or glutamine in all ecdysozoan GluCl sequences (Fig. 1b). Based on this conservation and our modelling, we therefore hypothesize that an arginine residue is responsible for coordinating the glutamate α-carboxyl at the “bottom” of the agonist-binding site in all GluCls; remarkably, in lophotrochozoan GluCls, this arginine comes from the opposite side of the intersubunit interface, some 65 amino acid positions downstream (see Fig. 1a) of the arginine in ecdysozoan GluCls.

### Arginine at either face of the agonist binding site is sufficient for glutamate recognition

If the presence of an arginine in either position were a valid basis for glutamate recognition, we reasoned that an arginine in *either* Loop A *or* Loop G should be sufficient for glutamate recognition. We tested this idea by mutating these positions in the *H. contortus* AVR-14B GluCl, and measuring glutamate-gated currents when expressed in *Xenopus laevis* oocytes. Before experimentally testing the role of the arginine at the bottom of the site, we sought experimental verification that glutamate binds to AVR-14B as predicted computationally.

**Figure 2:**
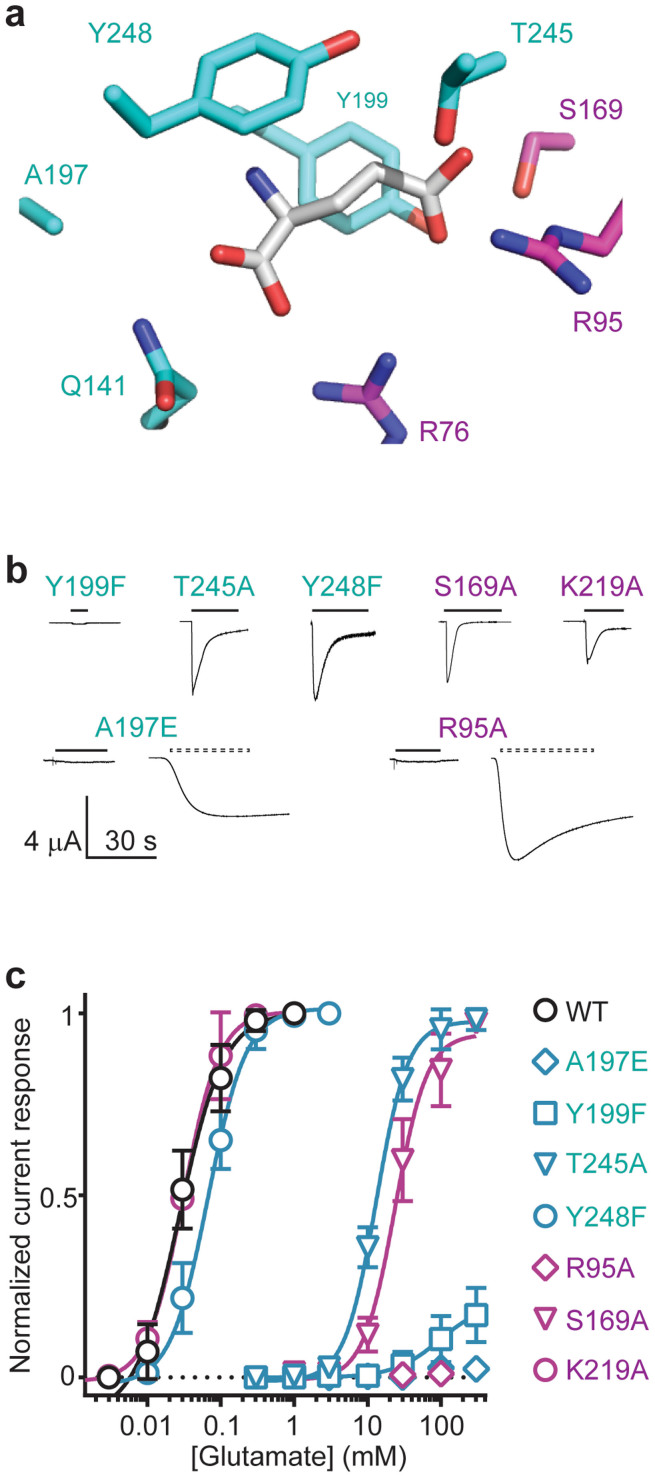
Mutations confirm a common binding mode of glutamate in ecdysozoan GluCls. (a) Close-up of computational glutamate docking to AVR-14B GluCl model, illustrating the agonist-binding side chains addressed in subsequent panels. (b) Exemplary glutamate-gated currents at oocytes injected with the indicated mutant AVR-14B cRNAs. Solid bars indicate glutamate application (1 mM for K219A and Y248F; 100 mM for all others); hashed bars indicate ivermectin (1 μM) application. (c) Averaged glutamate dose-response data. Mean (±s.e.m.) peak glutamate-gated currents were normalized to maximum glutamate-gated current (*n* = 3–7), except for R95A, A197E and Y199F (*n* = 3–6), which were normalized to averaged ivermectin-gated current amplitude (reported in Table 1). Data are fit with non-linear regression for illustration.

To this end, we mutated the AAA motif, generating Y199F (Loop B), T245A and Y248F (Loop C), R95A (Loop D) and S169A (Loop E) single-mutants. In our *in silico* docking, the phenyl rings of both Y199 and Y248 appear to sandwich the agonist amine, but the hydroxyl moiety of only Y199 is involved in direct interactions, both with the agonist γ-carboxyl and with the nearby S169 hydroxyl side chain (Fig. 2a). Accordingly, removal of these hydroxyl side chains via the Y199F and S169A substitutions dramatically decreased glutamate potency, whereas the Y248F substitution had no significant effect (Fig. 2b, c; Table 1). Our results also supported a principle role for the Loop D arginine in coordinating the agonist γ-carboxyl (Fig. 1b, c), as alanine substitution of R95 abolished and T245 decreased glutamate potency (Fig. 2b, c). An A197E mutation was designed to test the apparent requirement for glutamate recognition of a small, uncharged side chain at this position (initial residue in Loop B in Fig. 1b). This contrasts with a negatively charged glutamate residue at this position in GABA_A_Rs and GlyRs that is proposed to interact with the agonist primary amine, directly or via a water molecule^16^,^25^,^26^. Indeed, the A197E substitution abolished glutamate-gated currents (Fig. 2b, c), consistent with predictions that a glutamate residue at this position is better suited to GABA or glycine recognition^25^. Finally, K219A was designed to assess the possibility that this Loop F side chain (Fig. 1b) contributes to glutamate-binding, as is apparently the case in the *C. elegans* GLC-1 GluCl^18^. However, the K219A mutant showed similar glutamate sensitivity to WT (Fig. 2c; Table 1), indicating that this interaction is not a requirement. To verify that glutamate recognition, rather than structural integrity, was affected by these mutations, 1 μM ivermectin was shown to gate robust currents at glutamate-insensitive mutants (Fig. 2b; Table 1). In summary, these experiments point towards a common mode of glutamate recognition in ecdysozoan GluCls, where the agonist amine is sandwiched by the aromatic box and the γ-carboxyl is oriented towards Loop D of the complementary face.

**Figure 3:**
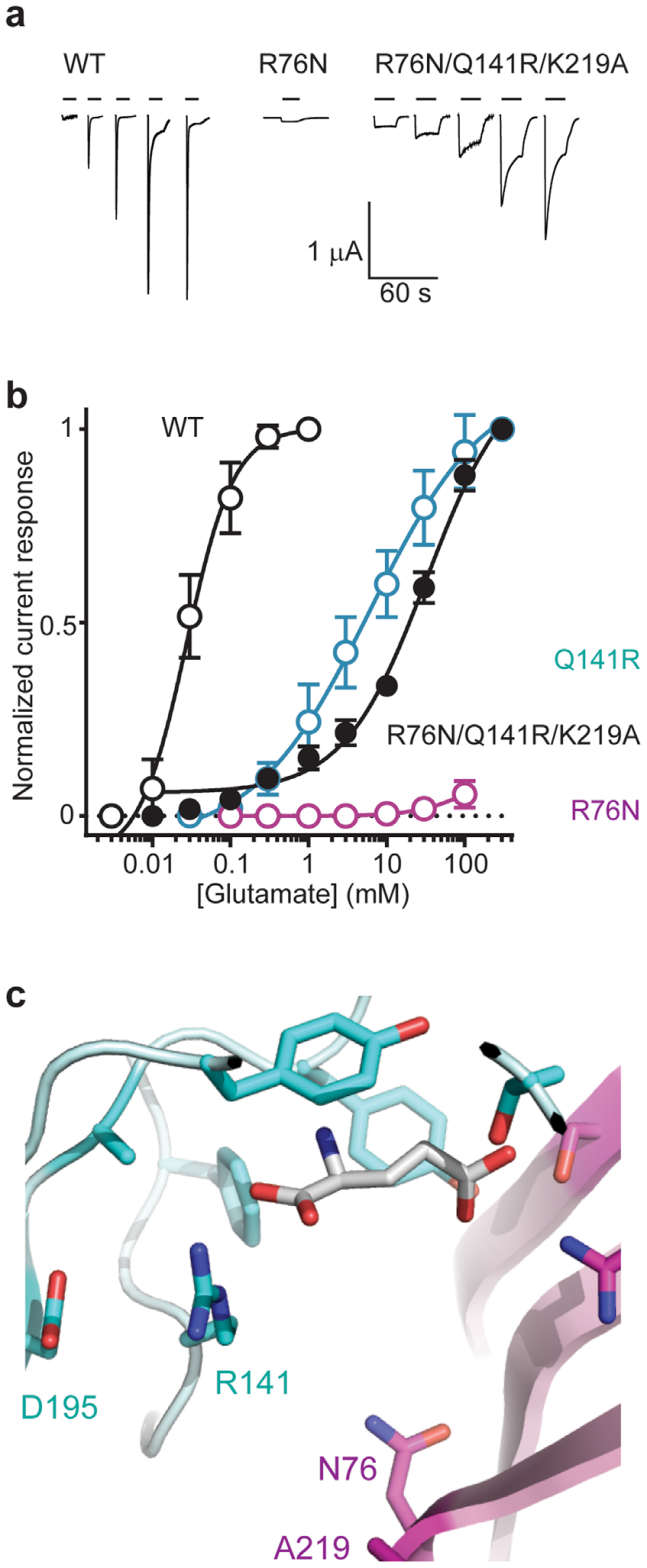
A principal *or* complementary subunit arginine recognizes the glutamate α-carboxyl in an ecdysozoan GluCl. (a) Glutamate gates robust currents at WT and triple-mutant R76N/Q141R/K219A but not at single-mutant R76N GluCls. Current responses to glutamate are shown for oocytes injected with WT or mutant cRNAs, as indicated. Bars indicate glutamate application (0.01, 0.03, 0.1, 0.3 and 1 mM for WT; 100 mM for R76N; 1, 3, 10, 30 and 100 mM for R76N/Q141R/K219A). (Scale bars refer to all experiments.) (b) Averaged glutamate dose-response data. Mean (±s.e.m.) peak glutamate-gated currents were normalized to maximum glutamate-gated current (*n* = 4–5), except for R76N (*n* = 6), which was normalized to averaged ivermectin-gated current amplitude (reported in Table 1). Data are fit with non-linear regression for illustration. (c) Close-up of computational glutamate docking to triple-mutant R76N/Q141R/K219A AVR-14B GluCl model. R141 ε and η nitrogen atoms are 3.2 and 3.0 Å from one agonist α-carboxyl oxygen atom. Only residues at the bottom of the site are labeled, including D195, just prior to Loop B; other residues appear as in Fig. 2a.

Finally, we addressed the ability of arginines at the bottom of the site to recognize the α-carboxyl of glutamate. To establish a “latent” GluCl without this ability, we generated the AVR-14B R76N mutant, replacing the Loop G arginine with a shorter, uncharged asparagine side chain, present in, for example, GlyR subunits (Fig. 1b). Although the R76N mutant was functionally expressed, as demonstrated by large ivermectin-gated currents (Table 1), it was not substantially gated by glutamate, even up to 100 mM (Fig. 3a, b), confirming the latency of the R76N GluCl. To then introduce a Loop A arginine, we made Q141R and K219A substitutions on the R76N background. Q141R introduces into Loop A the positively charged side chain present at this position in lophotrochozoan GluCls; the K219A mutation eliminates the possibility that in the absence of a Loop G arginine, this lysine residue could itself bind the glutamate α-carboxyl. This was apparently not the case for the R76N single-mutant, but we felt it necessary to remove this possibility, given the flexibility of lysine side chains in ligand binding^27^. At R76N/Q141R/K219A triple-mutant GluCls, glutamate gated robust currents that did not differ significantly from the original AVR-14B in terms of peak amplitude (Fig. 3a; Table 1), indicating that a Loop A arginine does indeed confer glutamate recognition on the latent GluCl.

Glutamate potency at this triple-mutant was, however, much less than at WT AVR-14B GluCls, as indicated by an EC_50_ value of 34 ± 3 mM (*n* = 5), almost three orders of magnitude greater than WT AVR-14B (Table 1). We also tested a GluCl with both Loop A and Loop G arginines. Notably, this Q141R single-mutant showed a similarly low glutamate potency (EC_50_ = 6 ± 1 mM; *n* = 5) to R76N/Q141R/K219A (Fig. 3b). Furthermore, both Q141R (*n*_H_ = 0.9 + 0.1) and R76N/Q141R/K219A (*n*_H_ = 0.9 + 0.1) were the only mutants tested that showed significantly decreased cooperativity, as indicated by lower Hill coefficients than WT (Table 1). We loosely interpret the similarities between these two mutants as evidence that, at least in the AVR-14B GluCl, a Loop A arginine side chain can “take over” the interaction with the glutamate α-carboxyl, irrespective of the presence of the Loop G arginine. Indeed, the two arginines within the Q141R receptor are likely to be mutually repulsive, and interactions with the negatively charged D195 residue (Fig. 3c) – present in AVR-14B but absent from SmGluCl-2 – may contribute to the fact that these mutant AVR-14B GluCls have lower glutamate affinity than WT AVR-14B or SmGluCl-2 GluCls. These data provide experimental support for our hypothesis that the presence of a principal face *or* a complementary face arginine residue forms the molecular basis for the convergent evolution of glutamate recognition.

### Phylogenetic analysis indicates polyphyletic origins of glutamate recognition

**Figure 4:**
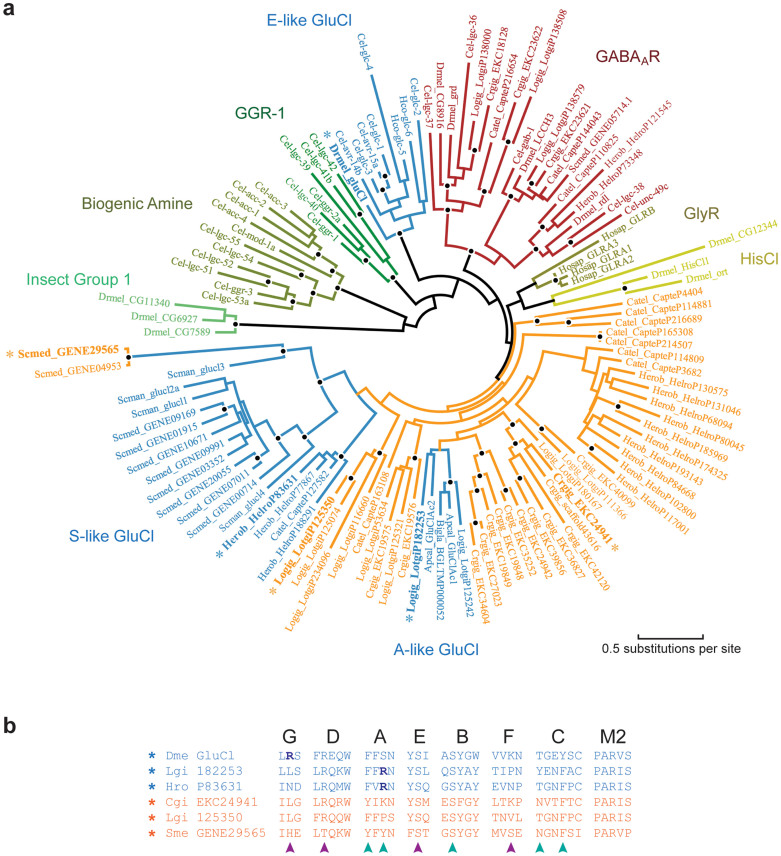
GluCl phylogeny. (a) Maximum likelihood phylogeny of anionic pLGIC subunit protein sequences representing the major clades identified from vertebrates and Ecdysozoa^2^, *S. mansoni* and *A. californica* GluCls, and newly identified, predicted anion channel subunits from the molluscs *C. gigas* and *L. gigantea*, the platyhelminth *S. mediterranea* and the annelida *C. teleta* and *H. robusta*. Major clades are identified in different colors and labeled, with sequences containing the glutamate-binding motif colored blue. Branch lengths are proportional to the number of substitutions per amino acid, and nodes with at least 90% SH and at least 70% bootstrap support are indicated by a black circle. Gene identifiers are from GenBank, and nematode gene names follow Beech *et al.*^52^. (b) Partial amino acid sequence alignment of selected subunits from (a). AAA motif and arginine residues are indicated by arrows as in Fig. 1b (cyan, principal face; magenta, complementary face). Note how orange sequences differ from verified GluCl subunits, in the absence of Loop G or A arginine residues, the presence of the initial Loop B glutamate residue, and/or the absence of the initial Loop C threonine residue. Dme, *Drosophila melanogaster*; Lgi, *Lottia gigantea*; Hro, *Helobdella robusta*; Cgi, *Crassostrea gigas*; Sme, *Schmidtea mediterranea*.

The origin of glutamate recognition in the pLGICs was previously unclear. There remained a possibility that the Ecdysosoan-like (E-like), Schistosome-like (S-like) and Aplysia-like (A-like) GluCls were paraphyletic, sharing a common ancestor that recognized glutamate, with receptors recognizing, for example, GABA or glycine also sharing this GluCl common ancestor. Here we can conclude that the E-like GluCls have a unique, monophyletic origin based on the fact that they coordinate glutamate binding through a Loop-G arginine that does not exist in the S-like or A-like GluCls. To determine whether the S-like and A-like GluCls are paraphyletic requires examination of the phylogenetic origin of the glutamate-binding motif that we have identified. The unrooted phylogeny in Fig. 4 highlights, in blue, the three distinct subunit clades that possess the glutamate-binding motif. Subunits from nematodes, insects and vertebrates representing previously identified major anionic pLGIC classes are also included^2^. A large number of predicted anionic pLGIC subunits can be identified in the recently available genomes of the gastropods, *Biomphalaria glabrata* and *Lottia gigantea*, the bivalve, *Crassostrea gigas*, the oligochaete *Capitella teleta*, the clitellate, *Helobdella robusta* and the turbellarian *Schmidtea mediterranea*. Many of these do not possess the AAA motif or the complete glutamate-binding motif and appear to represent several new subunit classes.

The A-like GluCl subunits form a monophyletic clade in the gastropods and are derived from subunits that would not have been able to bind glutamate, due to the absence of a crucial arginine and divergence from the AAA motif (Fig. 4). The relatively recent, derived position of the A-like GluCl subunits within the phylogeny confirms that the A-like and S-like GluCls converged on glutamate binding independently. The S-like GluCl clade is distinct and all subunits within share the AAA motif and Loop A arginine, with the exception of two *S. mediterranea* subunits that appear to have lost the Loop A arginine and are highly divergent from the other subunits (Fig. 4). All *S. mediterranea* subunits identified fall within the S-like GluCl clade, except for one GABA_A_R-like subunit, suggesting that glutamate is a major inhibitory neuromuscular transmitter of Platyhelminths. Of particular interest is a class of four subunits from the Annelida *C. taleta* and *H. robusta* that are within with the S-GluCl clade and share the lophotrochozoan glutamate-binding motif (e.g. HroP83631 in Fig. 4b). Current understanding of protostome taxonomy places Mollusca and Annelida as sister groups, descendent from a common ancestor that together with the Platyhelminthes form the Lophotrocozoa^28^. A shared S-like GluCl implies that the common ancestor of all Lophotrocozoa possessed this GluCl. The fact that to date no mollusk subunit has been identified that falls within this clade suggests that glutamate response mediated by the S-like GluCl was lost from the Mollusca. Whether this was before, after or coincident with appearance of the A-like GluCl is not clear. Analysis of this phylogeny shows three independent origins for glutamate recognition. We would expect that the S- and A-like GluCls emerged from glycine-binding subunits because the closely related subunits (orange in Fig. 4) that precede both these GluCls possess the initial Loop B glutamate residue conducive to glycine- and GABA-binding (see *Arginine at either face of the agonist binding site is sufficient for glutamate recognition*) and lack the closing Loop C arginine residue (Fig. 1b) required for high-affinity GABA-binding^15^,^29^.

## Discussion

There are precedents for convergent paths to agonist recognition in diverse pLGICs, although perhaps less conspicuous than the arrival of GluCls at glutamate recognition *via* opposite sides of the intersubunit interface. For example, cation-π interactions with the agonist amine are common to numerous pLGICs, but the interacting aromatic side chain is from Loop A in β2-containing GABA_A_Rs^30^, Loop B in ρ_1_ GABA_A_Rs^31^ and α1 GlyRs^17^, Loop C in GLC-2 GluCls^14^ and Loops B *and* C of RDL GABA_A_Rs^32^. Based on the position of the glutamate amine in our computational dockings, it seems that the main cation-π interaction may involve the Loop C aromatic of AVR-14B but the Loop B aromatic of SmGluCl-2. Together with results at *C. elegans* GLC-2 GluCls^14^, our docking tentatively suggests that the cation-π interaction may favour Loop C when the glutamate α-carboxyl is coordinated by a complementary face arginine. Perhaps more dramatic are the different means by which murine 5-HT-gated cation channels (5-HT_3_R) and *C. elegans* 5-HT-gated chloride channels (MOD-1) bind 5-HT. The amine of 5-HT forms a cation-π interaction with a Loop B tryptophan in the former and a Loop C tryptophan in the latter^33^,^34^, such that the same agonist probably binds in very different orientations in the two receptors^34^.

One limitation of our study is the focus on homo-pentameric receptors. This was required to accurately annotate putative GluCls in relation to verified GluCls, but as such, we may have excluded subunits that could, for example, form only the principal face of a glutamate-binding hetero-pentamer and thus lack the complementary face requirements for glutamate recognition. However, two lines of evidence suggest that our analysis, in which glutamate-binding principal and complementary faces are required for annotation as a GluCl, truly reflects the evolution of GluCls. First, most GluCls identified thus far form glutamate-gated homo-pentamers when expressed recombinantly (Fig. 1). Moreover, those subunits that form hetero-pentamers – additionally or exclusively – also possess both the principal and complementary subunit motives we have discussed^6^,^10^. Second, pharmacological and immunocytochemical experiments suggest that native GluCls occur as homomers and heteromers^35^,^36^, but all subunits contributing to these characterized native receptors – including AVR-14B employed in our experiments – contain the homo-pentameric glutamate recognition motif.

In summary, this study successfully employed a combination of computational, experimental and phylogenetic analyses to unravel the molecular basis of convergent evolution of glutamate recognition in pLGICs. This approach sheds new light on the origins of synaptic signalling and should prove similarly enlightening when applied to ligand-receptor interactions in other protein families. The finding that pLGICs recognizing glutamate have arisen independently at least three times suggests that care should be taken when inferring the pharmacological profile of ancestral receptors based solely on phylogeny. The suggestion that other pLGICs specific for acetylcholine^37^ and serotonin^34^ have arisen by convergent evolution implies that neurotransmitter signalling is more adaptable than has been appreciated before. In fact, the recent analysis of the ctenophore genome suggests that a neuromusculature regulated by glutamate may itself have multiple independent origins^4^.

## Methods

### Sequence alignments and computational dockings

The amino acid sequence alignment in Fig. 1 was performed in ClustalW2^38^ using sequences and agonist sensitivity information from original publications for *D. melanogaster* GluClα^5^ and RDL^39^,^40^, *M. domestica* GluClα^41^, *C. elegans* GLC-1 and −2^6^, GLC-3^42^ and AVR-15^43^, *H. contortus* GLC-5^44^, AVR-14B^45^ and UNC-49B^46^, *A. californica* GluCl1 and 2^9^, *S. mansoni* GluCl-2.1 and 3^10^ and *Homo sapiens* α1 GlyR^47^. Only portions of the ligand-binding loops were shown, in order to highlight the side chains that interact with ligands according to the GLC-1/glutamate crystal structure^18^, mutagenesis data^14^, and to our computational dockings. Portions of the channel-lining M2 helix were shown to illustrate the anion-selective motif of the channels^2^. Homology models of *H. contortus* AVR-14B, R76N/Q141R/K219A AVR14-14B and *S. mansoni* SmGluCl-2 were built on the *C. elegans* GLC-1/glutamate/ivermectin crystal structure (PDB 3RIF^18^) using Modeller^22^. Simulated glutamate docking to these models was performed with Autodock Vina^24^, allowing for a limited set of flexible side chains (those illustrated in Figure 1c except for the Loop F lysine; the two residues preceding the conserved Loop C threonine; and the residue two positions upstream of the Loop D arginine), within a 20 Å-sided cube encompassing the glutamate binding site. Agonist docked poses with the lowest predicted energy were selected for further analysis (shown in Fig. 1).

### Site-directed mutagenesis and electrophysiology

All mutant cDNAs were generated from AVR-14B cDNA in the T7 plasmid vector^45^ by conventional PCR with Pfu DNA polymerase (Thermo Scientific, Germany) and commercially generated mutagenesis primers (Eurofins MWG Operon, Ebersberg, Germany). All cDNAs were sequenced to confirm the presence of appropriate mutations (Eurofins MWG Operon) and linearized with XbaI (Thermo Scientific). cRNAs were synthesized with the mMESSAGE mMACHINE T7 Kit (Life Technologies GmbH, Darmstadt, Germany). *X. laevis* oocytes were used for two electrode voltage-clamp electrophysiology as previously described^48^ and under approval of the Technical University of Darmstadt (Agreement V54-19c20/15 DA8/Anz. 20). After 2–5 days, oocytes were perfused with extracellular solution (115 mM NaCl, 1 mM KCl, 1.8 mM CaCl_2_, 10 mM HEPES, pH 7.4 with NaOH) alone or containing agonist, and currents were sampled at 1 kHz and filtered at 500 Hz with a Digidata 1322A digitizer, Geneclamp 500B amplifier and Clampex software (Molecular Devices, Sunnyvale, CA, USA). Current responses to glutamate were plot against log glutamate concentration and fit with non-linear regression in Prism4 (GraphPad Software Inc., San Diego, CA, USA). Non-linear regression parameters were compared to the respective WT value with Student's unpaired t tests (Prism4).

### Phylogenetic tree

Genome data for *S. mediterranea* (v3.1), *C. gigas* (v9) and *C. teleta* (v1) were obtained from the Sanger Centre 50 Helminth Genome Initiative (ftp://ftp.sanger.ac.uk/pub/pathogens/es9/), *L. gigantea* and *H. robusta* from the EnsEMBL genome server (ftp://ftp.ensemblgenomes.org/pub) and *C. elegans* from WormBase^49^. Anionic pLGIC subunits were identified by tblastn using protein sequence from *C. elegans*, *S. mansoni* and *A. californica* and annotations were manually curated to maximize similarity between genes and organisms. Other anionic pLGIC sequences were obtained from GenBank. Protein sequences were aligned using MAFFT (http://mafft.cbrc.jp/alignment/software/), implemented in Geneious (v7.1.2)^50^. Sequence from the signal peptide, the intracellular loop and C-terminal tail that could not be aligned unambiguously were removed. Phylogeny reconstruction used PhyML (https://github.com/stephaneguindon/phyml-downloads/releases) as implemented in Geneious^51^, and significance of the internal tree branches was estimated using the SH-like statistics produced by PhyML and bootstrap resampling the dataset 100 times.

## Author Contributions

Study concept, design and preliminary bioinformatic analyses – T.L. Gene annotation and phylogenetic analysis – R.N.B. Homology modelling and computational dockings – B.A.C. Site-directed mutagenesis, electrophysiology and data analysis – T.L., M.M.J.L. and K.K. Manuscript preparation – T.L., R.N.B., B.A.C., A.J.W. and B.L.

## Figures and Tables

**Table 1:** Glutamate sensitivity of mutant AVR-14B GluCls

	Glutamate				Ivermectin^a^	
	EC_50_ (mM)	*n*_H_	*I*_max_ (μA)	*n*	*I*_max_ (μA)	*n*
Wild-type	0.04 ± 0.01	1.7 ± 0.2	3.1 ± 0.3	4	2.4 ± 0.4	5
R95A	b	b	0.04 ± 0.02^c^	3	4.8 ± 1.9	3
S169A	27 ± 4***	1.8 ± 0.2	4.3 ± 1.1	7		
Y199F	b	b	0.16 ± 0.04***	6	0.7 ± 0.2	2
T245A	16 ± 0.8***	2.0 ± 0.1	5.5 ± 0.8	7		
Y248F	0.07 ± 0.01	1.9 ± 0.2	4.3 ± 0.8	6		
R76N	b	b	0.19 ± 0.08^c^	6	3.6 ± 1.0	4
Q141R	6 ± 1**	0.9 ± 0.1**	1.1 ± 0.4**	5		
K219A	0.03 ± 0.01	2.1 ± 0.4	3.3 ± 0.5	3		
RN/QR/KA	34 ± 3***	0.9 ± 0.1**	2.3 ± 0.3	5		
A197E	b	b	0.01 ± 0.01^c^	6	2.4 ± 0.7	4

Mean ± s.e.m. from *n* experiments.

^*a*^1 μM ivermectin was applied to oocytes at which glutamate gated little or no currents (and to WT-expressing oocytes), in order to verify functional expression of GluCls.

^*b*^Could not be determined.

^*c*^Not significantly different from zero (one-sample t test).

**P < 0.01,

***P < 0.001, compared to WT (unpaired Student's t test).
